# Assessing the Influence of Socio-Demographic and Personal Traits (Knowledge, Attitude) on Practices Among Silica-Dust Exposed Mineworkers in the SADC Region

**DOI:** 10.3390/ijerph23060710

**Published:** 2026-05-27

**Authors:** Norman Nkuzi Khoza, Dingani Moyo, Phoka Caiphus Rathebe, Masilu Daniel Masekameni, Thokozani Patrick Mbonane

**Affiliations:** 1 Department of Environmental Health, School of Health Sciences, University of Johannesburg, Johannesburg 2028, South Africa; prathebe@uj.ac.za (P.C.R.); maseksd@unisa.ac.za (M.D.M.); 2 Human Capacity and Institutional Development, African Union Development Agency—NEPAD, Midrand 1685, South Africa; 3 Baines Occupational Health Services, Harare 0002, Zimbabwe; 4 Department of Community Medicine, Faculty of Medicine, National University of Science and Technology, Bulawayo ZW102105, Zimbabwe; 5 Developmental Studies, School of Social Sciences, University of South Africa, Pretoria 0003, South Africa

**Keywords:** silicosis, knowledge, attitude, practices, silica dust exposure, socio-demographics, mine, SADC, occupational health and safety

## Abstract

**Highlights:**

**Public health relevance—How does this work relate to a public health issue?**

- Exposed to crystalline silica dust remains a significant risk factor for irreversible, fatal chronic pulmonary disease such as silicosis, tuberculosis, lung cancer, and chronic obstructive pulmonary disease.

**Public health significance—Why is this work of significance to public health?**

- The study establishes a correlation between specific demographic characteristics, including age and educational background, knowledge, attitudes, and safe work practices. This correlation enables policymakers and health planners to tailor educational programs for workers who are at the highest risk of developing health-related conditions.
- The study covers the Southern African Development Community region, where fragmented regulatory frameworks and high exposure levels necessitate evidence-based, tailored, and locally relevant occupational health policies.

**Public health implications—What are the key implications or messages for practitioners, policy makers and/or researchers in public health?**

- Occupational health and safety practices are influenced by socio-demographic and personal traits (mine-related to occupational safety and health knowledge); therefore, these should be considered when designing and implementing occupational health and safety management programs in the mining industry.

**Abstract:**

Background: Exposure to respirable crystalline silica dust remains a significant occupational health challenge in the Southern African Development Community region, leading to high incidences of silicosis and pulmonary tuberculosis, particularly among mining workers. This study evaluated the knowledge, attitudes, and practices (KAP) of mineworkers regarding silica dust risks across Lesotho, Malawi, Mozambique, and Zambia. Methods: A cross-sectional analytical study was conducted involving 1440 mineworkers exposed to silica dust in mines across four SADC countries. Data were collected using structured questionnaires covering socio-demographic traits and knowledge, attitude, and practices. Data analysis was conducted using Stata version 18. Results: While 91% of participants exhibited adequate knowledge and 88% demonstrated acceptable practices, 51% maintained negative safety attitudes. Knowledge scores were positively correlated with company training (*r* = 0.386). However, a “Training Paradox” emerged in the regression model: compulsory company training was significantly associated with a 1.20-unit decrease in practice scores, whereas external training and higher education levels (+2.98 units) predicted improved compliance. Technicians and younger workers were identified as higher-risk cohorts. Conclusions: The findings suggest that top-down mandatory training may trigger psychological reactance, undermining behavioural safety. To mitigate silica-related diseases, industry stakeholders should transition toward participatory, role-specific safety interventions that prioritize worker autonomy and cognitive engagement over administrative compliance.

## 1. Introduction

Chronic inhalation of silica dust is the primary cause of silicosis, a permanent and incurable fibrotic lung disease, and significantly increases the risk of pulmonary tuberculosis (TB), lung cancer, and other respiratory impairments [1,2]. While international standards such as those set by OSHA have established permissible exposure limits (PEL), many workers in resource-constrained settings continue to be exposed to concentrations well above these safety thresholds [3,4].

The Southern African Development Community (SADC) region is historically characterised by a high burden of occupational lung diseases due to its extensive mining history and migrant labour systems [5,6,7,8,9]. In countries like South Africa, Zambia, and Lesotho, the prevalence of silicosis among mineworkers remains a critical public health priority, often exacerbated by the biological interaction between silica exposure, TB, and HIV [9,10,11,12,13,14,15,16]. A study of a small-scale tanzanite mine in Tanzania reported that miners experienced obstructive and restrictive lung diseases, particularly those who had worked in the mining industry for more than 10 years [17]. Recent data indicate that, despite stringent national occupational health acts, many workers, especially in artisanal and small-scale mining (ASM), lack access to adequate healthcare and protective measures [18,19,20].

Effective management of silica exposure relies not only on engineering controls but also on the individual behavioural compliance of mineworkers [21]. Scientific evidence suggests that while some miners possess basic knowledge of dust-related risks, there are significant gaps in understanding the long-term, incurable nature of silicosis and the necessity of specialised personal protective equipment (PPE) [22,23]. Poor attitudes, such as the perception that minor accidents are “part of the job” or that health and safety rules are biased, can further hinder the adoption of safe work practices [22,24,25,26,27].

There is a growing recognition that socio-demographic traits (factors) such as age, education level, work experience, and income significantly influence safety practice in the mining sector [28,29]. For instance, studies have shown that workers with higher education levels are more likely to be aware of the adverse effects of silica, while younger workers with less experience may be at a higher risk for occupational injuries [30,31]. In the SADC region, factors such as migrant status and housing conditions also contribute to the overall health and safety of the mining workforce [32,33].

Despite the established links between socio-demographic traits and occupational health outcomes, there is limited comprehensive research specifically assessing how these variables influence the knowledge, attitudes, and practices (KAP) of silica-dust-exposed workers across the diverse SADC region [15,34,35]. Understanding these determinants is crucial for developing targeted educational programs and evidence-based interventions that go beyond technical controls to address human behavioural factors. This study aims to assess the influence of socio-demographic traits on knowledge, attitudes, and practices among silica-dust-exposed mineworkers in the SADC region. Furthermore, assess the influence of personal traits (individual knowledge and attitude) on safety practices among workers exposed to silica dust. By identifying specific demographic predictors of poor KAP scores, this research seeks to inform regional policy and workplace health promotion strategies to eliminate silicosis and reduce the burden of occupational lung diseases.

Despite rigorous safety protocols, silica-dust exposure continues to drive high rates of silicosis and TB across the SADC mining sector [36]. While technical controls exist, worker compliance remains inconsistent, often falling below desired compliance standards in high-risk zones [36,37]. A critical gap remains in understanding how socio-demographic traits, such as age, education, and migrant status, shape a worker’s knowledge, attitude, and practices (KAP) regarding dust safety. Current regional health strategies typically use a “one-size-fits-all” approach, failing to account for how individual backgrounds influence risk perception and PPE usage [30,35]. Without identifying these specific demographic drivers, interventions remain generic and ineffective. The study evaluated the influence of socio-demographic factors on the KAP of mineworkers exposed to silica dust. Ultimately, these findings serve to guide the development of targeted, region-specific safety policies within SADC. Specifically, the study sought to: (i) evaluate the current levels of knowledge, attitudes, and safety practices regarding silica dust; (ii) determine the correlation relationship between socio-demographic factors and knowledge and attitude in the current study, and (iii) determine the statistical relationship between practices with socio-demographic factors, knowledge and attitude among mines in the SADC region.

## 2. Materials and Methods

### 2.1. Study Design, Site and Setting

The study adopted a cross-sectional analytical method to achieve its aim and objectives. This study was conducted as part of the Southern Africa TB and Health Systems Support project. It was conducted in four countries, namely Lesotho, Malawi, Mozambique and Zambia.

### 2.2. Study Population and Sampling Strategy

The study focused on mine workers who were or might have been exposed to crystalline silica dust in open-cast or underground mines, including those mining diamonds, sandstone, and operating quarries. Full-time and long-term contract workers were included as study participants, while seasonal workers and those who did not provide consent were excluded. The study employed purposive sampling, with researchers specifically identifying and targeting mines where workers might be exposed to crystalline silica dust and obtaining permission from gatekeepers. Subsequently, workers were recruited to participate in the study, and informed consent was requested before their participation.

### 2.3. Questionnaire

A self-administered structured questionnaire was used to collect data for this study. It consisted of sections on socio-demographic information of the participants and personal traits such as knowledge of occupational health and safety in mines, including related policies, risk and prevention strategies, attitudes towards mine safety, work incidents, related health outcomes, and practices concerning occupational health and safety [22]. The questionnaire was piloted with 80 mine workers (20 from each country) who were not included in the actual study. It was tested for internal consistency reliability, achieving a score of 0.8, indicating good or adequate reliability. (good or adequate) was achieved [38].

Socio-demographics information included gender (female/male), age categorised into five groups (21–30 years old/31–40 years old/41–50 years old/51–60 years old/61 years old and older), country of origin (Lesotho/Malawi/Mozambique/Zambia), marital status (single/married/widowed), educational level (no schooling/primary school/high school/tertiary), job description (general worker/driver/plant operator/technician/supervisor), year of experience in a mine (1–10 years/11–20 years/21 years or more), received general/external mine occupational health and safety training (no/yes) and company or compulsory mine occupational health and safety training (no/yes).

The study participants were asked 15 questions on knowledge; these were yes-or-no questions. The “yes” response was considered a correct answer and allocated a score of 1, while “no” as incorrect answer (with zero score allocated). The knowledge had a potential total score of 0–15 per individual after adding the score of each question. A score of 9 or above indicated adequate knowledge, while a score below 9 indicated inadequate knowledge of silica dust and its risks and prevention. The questions on knowledge are shown in Table A1 (Appendix A).

The attitude section consisted of nine questions, each with two response options: “disagree” or “agree,” as illustrated in the Table 1 below. A value of 0 was assigned to the response “disagree”, and a value of 1 to the response “agree.” The scores for each question were subsequently aggregated to generate an overall attitude score, which ranged from 0 to 9. A total score of 4 or below indicated a negative attitude, whereas a score of 5 or above reflected a positive attitude.

The dependent variable of the study, referred to as the study outcome, was the practice score. This practice score was derived from a set of 14 items, each assessed with binary responses (yes = 1 or no = 0), where a “yes” response represented the correct answer and a “no” response indicated an incorrect answer, as illustrated in Table 2. The responses of each participant were combined to produce a total score ranging from 0 to 14. A score below 7 indicated unacceptable practice behaviour, while a score of 7 or above indicated acceptable practice behaviour.

### 2.4. Data Analysis

The collected data were entered into Microsoft Excel for cleaning and coding and subsequently transferred to Stata version 18 (StataCorp, College Station, TX, USA) for descriptive and inferential analysis. First, descriptive data were presented using tables that displayed frequencies and percentages for categorical variables, including age, country of origin, marital status, educational level, job description, years of experience in a mine, and types of training received (general/external mine occupational health and safety training and company or compulsory mine occupational health and safety training). The continuous variables (knowledge, attitude, and practice scores) in this study were reported as means with standard deviations (SDs) and ranges.

Pearson correlation was performed to assess the direction and strength of the relationships between socio-demographic variables and personal traits (knowledge and attitude scores), with results presented in a heatmap correlation matrix. A *p*-value of 0.05 was used to indicate significant relationships. Lastly, linear regression was employed to determine factors influencing the practice score. A bivariate linear regression was conducted between the practice score (dependent variable) and each independent variable (socio-demographics and personal traits). In the final model, a multiple linear regression analysis was performed using a backward stepwise approach to identify socio-demographics and personal traits influencing practices. The findings were presented in a table displaying the β coefficients, standard error (std err), *p*-values (with 0.050 representing statistical significance), and 95% confidence intervals.

## 3. Results

### 3.1. Study Population’s Socio-Demographics

The study involved 1440 participants, the majority of whom were males (n = 1295; 90%), with fewer females (n = 145; 10%). Participants’ ages ranged from 21 to over 61 years, with 595 (45%) falling between 31 and 40 years old. Table 3 (shown below) provides a detailed description of the study participants. The study was conducted across four countries, with the largest group from Lesotho (n = 385; 27%) and the smallest from Malawi (n = 315; 22%). There were more married participants (n = 826; 58%) than those who were single (n = 595; 41%) or had lost a partner (n = 19; 1%). Additionally, 730 (51%) participants had a high school education, while others had no formal schooling (n = 403; 28%), completed primary school (n = 173; 12%), or attained tertiary education (n = 134; 9%). More participants had done general/external OHS mine-related training (n = 1374, 95%) and compulsory company OHS training (n = 1170, 81%).

### 3.2. Safety Knowledge, Attitude and Practices of the Study Participants

**Figure 1:**
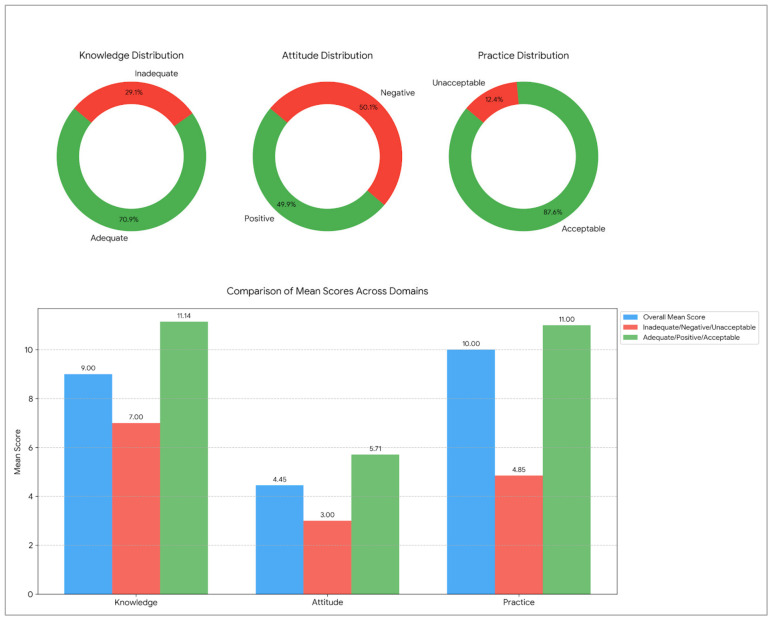
Distribution of knowledge, attitude, practices and their scores.

The overall safety knowledge was classified into two categories: adequate and inadequate, as shown in Figure 1. Among the participants, 21% (n = 419) demonstrated inadequate knowledge, with a mean score of 7 (SD = 1.22), ranging from 4 to 8. In contrast, 91% (n = 1021) exhibited adequate knowledge, achieving a mean score of 11.14 (SD = 1.58) regarding occupational health and safety in the context of silica dust mining environments. The overall mean knowledge score in this study was 9 (SD = 2.9), with scores ranging from 4 to 14.

The overall mean attitude score in the study was 4.45 (SD = 1.51), with scores ranging from 0 to 8. A majority of the participants (n = 722, 51%) exhibited a negative attitude toward the safety environment in their workplace, yielding a mean attitude score of 3 (SD = 0.90), with scores ranging from 0 to 4. Conversely, less than half of the participants demonstrated a positive attitude, reflected in a mean attitude score of 5.71 (SD = 0.78), with scores ranging from 5 to 8.

The majority of participants (n = 1261, 88%) demonstrated acceptable practice, evidenced by a mean practice score of 11 (SD = 2.30), with scores ranging from 7 to 14. Conversely, participants categorised as exhibiting unacceptable practices (n = 179, 12%) obtained a mean practice score of 4.85 (SD = 1.50), with scores ranging from 0 to 8. Overall, the study’s mean practice score was 10 (SD = 3.02), with scores spanning from 0 to 14.

### 3.3. Correlation Between Socio-Demographics and Knowledge and Attitudes Scores

**Figure 2:**
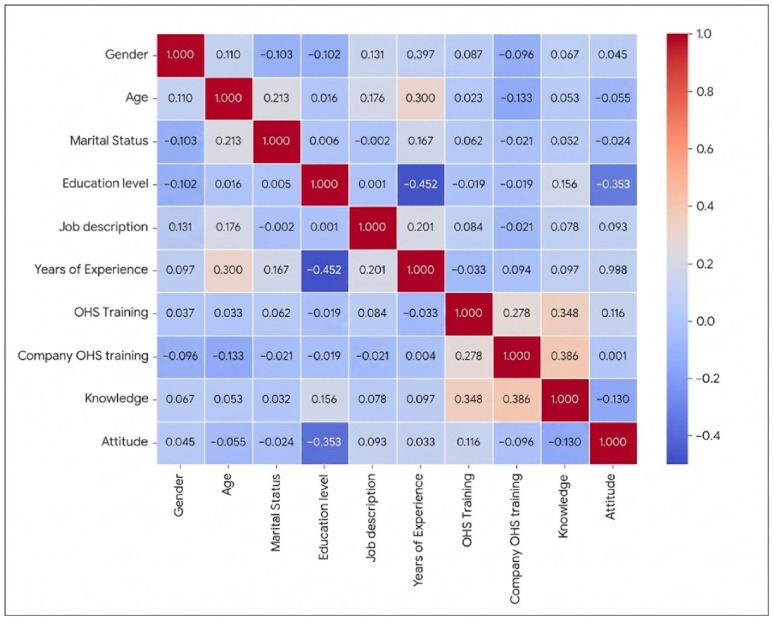
Heatmap correlation matrix between knowledge and attitude with socio-demographic factors.

Figure 2 presents a heatmap correlation matrix detailing the socio-demographic variables significantly correlated with knowledge and attitude. The knowledge score showed a positive significant correlation with gender (*r* = −0.067; *p* < 0.011), age (*r* = 0.053; *p* = 0.044), educational level (*r* = 0.156; *p* < 0.001), job description (*r* = 0.078; *p* = 0.003), years of experience (*r* = 0.097; *p* < 0.001), general/external mine-related OHS training (*r* = 0.348; *p* < 0.001), and compulsory company OHS training (*r* = 0.386; *p* < 0.001).

The attitude score demonstrated a significant positive correlation with job description (*r* = 0.093; *p* < 0.001), years of experience (*r* = 0.098; *p* < 0.001), and general/external mine-related OHS training (*r* = 0.116; *p* < 0.001). Conversely, it exhibited a significant negative correlation with age (*r* = −0.055, *p* = 0.038), education (*r* = −0.353, *p* < 0.001), and knowledge score (*r* = −0.130, *p* < 0.001).

### 3.4. Factors Influencing Practice Scores in the Study

In the bivariate linear regression (shown in Table 4), practice score had a significant positive association with age (41 to 50 years old: *p* < 0.001; 51 to 60 years old: *p* < 0.001), marital status (being married: *p* < 0.001), educational level (having primary school education: *p* < 0.001; high school certificate: *p* < 0.001; tertiary certification: *p* < 0.001), job description (being a plant operator: *p* = 0.002; being a supervisor: *p* = 0.001), having attended general/external mine-related OHS training (*p* < 0.001), and knowledge score (*p* < 0.001). Conversely, it had a significant negative association with being a technician (job description: *p* = 0.002), having 10–20 years of work experience (*p* = 0.033), having attended company or compulsory OHS training (*p* = 0.029), and attitude score (*p* < 0.001).

The final regression model in Table 4 demonstrates an R-squared value of 0.0931 and an adjusted R-squared value of 0.0925, along with a root mean square error (RMSE) of 2.8737. The details are displayed in Table 4 below. The final model indicates that an increase in age (41 to 50 years old: *p* = 0.012; 51 to 60 years old: *p* = 0.005) is associated with a statistically significant increase in practice scores, with estimated average increases of 0.60 and 0.95 units, respectively. Being married and having attended general or external mine-related OHS training were significantly associated with increases of 0.32 and 1.28 units, respectively, in practice scores. Additionally, a higher educational level was significantly linked to increases in practice scores: an increase of 2.16 units for those with primary school education, 1.98 units for those with a high school certificate, and 2.98 units for those with a tertiary certification. Furthermore, having worked for 11 to 20 years in the mining industry was associated with a 0.58 unit increase in practice scores. Lastly, an increase in knowledge scores was linked to a 1.84 unit increase in practice scores. However, being a technician and having attended compulsory or company OHS training had significant negative associations with practice scores, resulting in decreases of 1.23 and 1.20 units, respectively.

## 4. Discussion

This study critically assessed the knowledge, attitudes, and practices of mineworkers regarding occupational exposure to respirable crystalline silica dust across four Southern African countries. The results reveal important insights into persistent gaps in occupational health education and behavioural adherence despite known health risks, thereby aligning with prior findings in the literature on silica dust exposure hazards and control challenges in mining communities. This study included more males than females, indicating that the industry is still male-dominated, as reported elsewhere [39]. It was encouraging to see that more participants had received both general/external OHS mine-related training and compulsory company OHS training. These trainings are not just a tick-box exercise; they are essential for preventing fatalities, serious occupational incidents and injuries [35,40,41]. Knowledge scores among participants exhibited considerable variation; the majority demonstrated a satisfactory level of awareness, while a significant minority displayed insufficient understanding. This finding aligns with concerns articulated raised by other researchers regarding deficiencies in worker education programs and the necessity for continuous training reinforcement [42].

The significant correlations observed between knowledge and socio-demographic factors, such as gender, age, educational level, years of experience, general/external mine-related OHS training (they might have completed this training before being employed by the mine) and compulsory company OHS training highlight the complex interplay of individual and contextual elements influencing silica risk awareness. The findings suggest that formal training programs function as the principal mechanism for hazard recognition and risk mitigation [43,44]. This observation is consistent with recent industry trends, wherein integrated, evidence-based training models have been recognized for their substantial impact on reducing fatalities in the mining sector [22,28,29]. The marginally higher correlation for company-specific training compared to external training (0.386 vs. 0.348) may underscore the significance of context-specific knowledge, suggesting that employees derive greater benefit from understanding the unique geological and operational risks associated with their specific worksite rather than from generalized safety theories. Moreover, the positive correlations observed between knowledge and both years of experience and age imply a “cumulative learning” effect [45]. While younger or less experienced workers may display elevated risk-taking tendencies due to overconfidence, older employees tend to cultivate a more nuanced understanding of hazards through prolonged exposure [29,46]. However, the relatively low correlation coefficients for age indicate that, although experience contributes to safety awareness, it cannot replace the necessity of formal, up-to-date training [45].

The significant correlation between knowledge and educational level highlights a potential “literacy gap” in safety communication. Workers with higher levels of formal education may find it easier to comprehend complex safety documentation and legislative requirements [47]. This observation is particularly salient, as prior research has identified low educational attainment and linguistic diversity as considerable barriers to effective safety training within the South African mining context [48,49]. Additionally, the significant correlations between knowledge and both gender and job description suggest that safety knowledge is also delineated by role and gendered participation within the workforce. In numerous heavy industries, men and women are often assigned to disparate operational roles, which influences their exposure to specific safety protocols and training cycles [48,50,51,52]. For example, administrative or specialized technical positions frequently receive different levels of OHS instruction compared to frontline mineworkers.

Attitudinal analysis reveals that positive safety attitudes among mine workers are primarily influenced by job description, years of experience, and generic/external OHS training. Conversely, age, education levels, and knowledge scores demonstrate significant negative correlations with safety attitudes, suggesting a disconnect in which theoretical knowledge does not effectively translate into safety compliance. The significant negative correlation observed between compulsory company OHS training and both knowledge and practice scores can be characterized as the ‘Training Paradox’ [36,53].’ This finding indicates that when safety education is perceived as a mandatory, top-down administrative requirement, it may inadvertently provoke psychological reactance among miners [54,55]. In these contexts, training is often regarded not as a resource for self-protection but as a punitive or superficial ‘tick-box’ exercise aimed at shifting corporate liability rather than genuinely mitigating risk [56]. This fosters a culture of ‘safety fatigue,’ where the repetitive and disjointed nature of the curriculum leads to cognitive disengagement. Consequently, although workers may technically attend the training sessions, the perceived lack of relevance and the loss of autonomy result in lower knowledge retention and a disregard for recommended safety practices [54,55]. To address this trend, industry stakeholders must shift from high-frequency, passive mandates to participatory, scenario-based learning that empowers workers as active contributors to the safety culture, rather than as passive recipients of bureaucratic instruction.

A significant trend identified in the final model is the positive relationship between age and safety compliance. Workers in the 41 to 50 and 51 to 60 age groups demonstrated increases in practice scores. This age-dependent enhancement suggests that senior workers possess a deeper understanding of risk, likely informed by years of observing the consequences of safety failures [57]. This assertion is further supported by the increase in practice scores among miners with 11 to 20 years of industry experience. Additionally, marital status (being married) was found to positively influence practice scores, potentially indicating that workers with substantial family responsibilities adopt a more risk-averse profile, perceiving safety practices as essential for ensuring long-term household stability.

The final regression analysis model highlights a robust linear relationship between formal education and safety implementation. Practice scores showed significant improvement across all educational levels (primary education, high school certificate, tertiary certification). This trend suggests that higher educational attainment enhances cognitive capacity to process complex safety protocols and apply them effectively in dynamic environments. Notably, an increase in knowledge scores was associated with an increase in practice scores, reaffirming the fundamental principle that while knowledge alone does not guarantee safety, it is a critical prerequisite for appropriate practice [36,53,58]. A key finding of this study is the divergent impact of various training modalities. General or external mine-related OHS training was linked to an increase in practice scores. In contrast, compulsory company OHS training was significantly associated with a decrease in practice scores. This disparity suggests that external training may be perceived as more objective or valuable, whereas internal, mandatory sessions may be subject to “institutional fatigue.” When training is framed as a repetitive, top-down administrative obligation, it can lead to psychological reactance, wherein workers may attend physically but disengage cognitively, resulting in a net negative effect on their actual field behavior [53,54,59].

Finally, the final regression model indicated that being a technician resulted in a decrease in practice scores. This may be attributable to the specific demands of technical work, which often involves high-pressure troubleshooting or maintenance tasks that prioritise production over adherence to standard operating procedures. This finding underscores the need for targeted, role-specific safety interventions that address the unique pressures inherent in the responsibilities of technicians, who represent a high-risk cohort.

The study’s primary strength is its robust multi-country design, utilizing a sample across four Southern African nations to provide an overview of silica-related health and safety practices. Additionally, the use of a validated instrument and multivariate regression analysis enables precise identification of the “Training Paradox” and other socio-demographic determinants of safety behaviour. Limitations of the current research include the reliance on self-reported data, which may introduce biases, and the cross-sectional design that precludes causal inferences. Although the study was conducted in four countries, the use of purposive sampling and the exclusion of seasonal workers may restrict the generalizability of the findings to the wider informal or temporary mining workforce in Southern Africa. Lastly, the study did not include objective environmental exposure measurements (e.g., personal dust sampling for respirable crystalline silica). Consequently, the reported Knowledge, Attitudes, and Practices (KAP) could not be correlated with actual exposure levels to validate the clinical risk environment.

Future research should incorporate longitudinal designs, objective exposure assessments, and intervention studies to establish evidence-based strategies aimed at reducing the burden of silica-related occupational diseases in this region.

## 5. Conclusions

This study provides a comprehensive analysis of the factors influencing OHS knowledge, attitudes, and practices (KAP) regarding silica dust exposure among mineworkers in Southern Africa. A critical finding is the identified “Training Paradox”; while general external training significantly enhanced safety practices, compulsory company-led training was associated with a decrease in practice scores, likely attributable to psychological reactance and institutional fatigue. Additionally, although the majority of workers demonstrated adequate knowledge (91%) and acceptable practices (88%), over half (51%) maintained a negative attitude toward the safety environment. The regression model highlights educational attainment and age as primary determinants of safety compliance, while technicians emerge as a high-risk group with lower practice scores. These findings necessitate a strategic shift from passive, top-down mandates to participatory, scenario-based interventions. Future OHS policies must bridge the gap between theoretical knowledge and behavioral adherence by addressing role-specific pressures and fostering an inclusive, autonomy-supportive safety culture to effectively mitigate occupational lung diseases.

## Figures and Tables

**Table 1:** Questions on attitudes.

No	Question	Variable
Q1	Do you believe that our work is meant for the brave and not for people who are worried about Health & Safety?	H & S concern
Q2	Do you believe that minor accidents are a normal part of my daily work?	Minor accidents
Q3	Do you believe that health and safety laws are biased against small-scale miners?	H & S laws
Q4	Do you believe that it is difficult to follow health and safety procedures?	H & S procedure
Q5	Do you believe that workers react strongly against other workers who break health and safety rules?	H & S rules
Q6	Do you feel safe in my work environment?	Work environment
Q7	Do you believe that mine dust is the cause of diseases such as pneumoconiosis, silicosis, lung cancer and mesothelioma?	Diseases
Q8	Do you believe that high levels of dust exposure in mines could be a cause of occupational lung diseases amongst mineworkers?	Dust exposure
Q9	Do you believe that some ex-mineworkers got ill or died due to exposure to crystalline silica or mine dust?	Ill health or death

**Table 2:** Questions on practices in this study.

Question	Response
1. Is there evidence of a health and safety implementation strategy in my company?	(no/yes)
2. Do you hold health and safety meetings regularly?	(no/yes)
3. Does your employer conduct dust and noise surveys at regular intervals?	(no/yes)
4. Does the employer conduct individual risk assessments before commencing your duties as an employee?	(no/yes)
5. Is there a health and safety representative in my work area?	(no/yes)
6. Taking air measurements for dust at the mine is good practice for controlling dust exposures?	(no/yes)
7. Spraying water/chemical agents on travel/haul roads is an effective way to reduce dust levels?	(no/yes)
8. Is keeping the dump truck’s cabin door closed a good practice to keep dust levels low inside?	(no/yes)
9. Do you wear dust/paper mask is important when working in dusty areas?	(no/yes)
10. Is wearing a dust/paper mask important when working with chemicals, gases or vapours?	(no/yes)
11. Can you protect your hands from workplace hazards and risks by wearing good protective gloves?	(no/yes)
12. Does following approved re-entry times after blasting limit your exposure to dust and blasting fumes/gases?	(no/yes)
13. Does wearing hearing protection (earplugs/earmuffs) at work protect you from hearing loss?	(no/yes)
14. In the past 12 to 24 months, have you participated in any health and safety awareness training sessions?	(no/yes)

**Table 3:** Study participants’ socio-demographic variables distribution.

Socio-Demographic Variables	Frequency (n)	Percentage (%)
Gender		
Female	145	10%
Male	1295	90%
Age		
21–30 years old	542	37%
31–40 years old	595	41%
41–50 years old	201	14%
51–60 years old	96	7%
61 years old and older	6	1%
Country of Origin (CO)		
Lesotho	385	27%
Malawi	315	22%
Mozambique	384	27%
Zambia	356	24%
Marital status		
Single	595	41%
Married	826	58%
Widowed	19	1%
Highest Educational level		
No schooling	403	28%
Primary school	173	12%
High school	730	51%
Tertiary	134	9%
Job description (JOB)		
General worker	655	45%
Driver	53	3%
Plant operator	554	39%
Technician	95	7%
Supervisor	83	6%
Years of experience (Experience)		
1–10 years	721	50%
11–20 years	684	48%
21 years or more	35	2%
OHS training		
No	66	5%
Yes	1374	95%
Company OHS training		
No	270	19%
Yes	1170	81%

**Table 4:** Multivariate Analysis: Final model on the risk factors for practices.

Variables		Bivariate Analysis			Multivariate Analysis		
		Coefficient (Std Err)	*p*-Value ^a^	95% Conf. Interval	Coefficient (Std Err)	*p*-Value	95% Conf. Interval
Gender	Female	Ref					
	Male	0.04 (0.26)	0.880	−0.48 to –0.56	−0.03 (0.26)	0.916	−0.53 to 0.48
Age	21 to 30 years old	Ref					
	31 to 40 years old	0.18 (0.18)	0.306	-0.16 to 0.52	0.08 (0.17)	0.630	−0.25 to 0.42
	41 to 50 years old	1.57 (0.24)	<0.001 *	1.09 to 2.04	0.60 (0.24)	0.012 *	0.13 to 1.07
	51 to 60 years old	1.62 (0.33)	<0.001 *	0.98 to 2.27	0.95 (0.33)	0.005 *	0.29 to 1.60
	61 years old and older	0.61 (1.21)	0.614	−1.77 to 3.00	−1.34 (1.20)	0.263	−3.69 to 1.01
Marital Status	Single	Ref					
	Married	0.71 (0.16)	<0.001 *	0.39 to 1.03	0.32 (0.16)	0.043 *	0.01 to 0.63
	Widowed	0.33 (0.70)	0.635	−1.04 to 1.70	0.00 (0.70)	0.100	−1.37 to 1.37
Education	No schooling	Ref					
	Primary school	2.10 (0.26)	<0.001 *	1.59 to 2.61	2.16 (0.35)	<0.001 *	1.47 to 2.84
	High school	2.02 (0.18)	<0.001 *	1.67 to 2.37	1.98 (0.24)	<0.001 *	1.52 to 2.45
	Tertiary	2.51 (0.29)	<0.001 *	1.95 to 3.07	2.98 (0.34)	<0.001 *	2.32 to 3.64
Job Description	General worker	Ref					
	Driver	−0.46 (0.43)	0.276	−1.30 to 0.37	−0.87 (0.40)	0.029 *	−1.65 to −0.09
	Plant operator	0.52 (0.17)	0.002 *	0.19 to 0.86	0.09 (0.17)	0.615	−0.25 to 0.42
	Technician	−1.01 (0.33)	0.002 *	−1.65 to −0.37	−1.23 (0.31)	<0.001 *	−1.83 to −0.64
	Supervisor	1.18 (0.35)	0.001 *	0.50 to 1.87	0.34 (0.33)	0.311	−0.32 to 0.99
Experience	1–10 years	Ref					
	11–20 years	−0.34 (0.16)	0.033 *	−0.66 to −0.03	0.58 (0.24)	0.008 *	0.15 to 1.01
	21 years or	0.01 (0.52)	0.994	−1.02 to 1.03	−0.02 (0.54)	0.976	−1.08 to 1.05
OHS Training	No	Ref					
	Yes	1.96 (0.38)	<0.001	1.22 to 2.70	1.28 (0.37)	<0.001 *	0.55 to 2.01
Company OHS Training	No	Ref					
	Yes	−0.44 (0.20)	0.029 *	−0.84 to −0.05	−1.20 (0.20)	<0.001 *	−1.60 to −0.80
Knowledge	Not Adequate	Ref					
	Adequate	2.03 (0.17)	<0.001 *	1.70 to 2.35	1.84 (0.18)	<0.001 *	1.48 to 2.19
Attitude	Negative	Ref					
	Positive	−0.80 (0.16)	<0.001 *	−1.11 to −0.49	0.26 (0.16)	0.107	−0.06 to 0.58

^a^ Statistical significance *p*-values set at 0.050, * shows a statistically significant *p*-value, F(1.1438) = 147.67, Prop > F < 0.0001, R-squared = 0.0931, Adj R-squared = 0.0925, Root MSE = 2.8737.

## Data Availability

The data can be accessed by contacting the authors based on a reasonable/justifiable request and in adherence to the South African Protection of Personal Information Act 4 of 2013 (POPIA).

## Abbreviations

The following abbreviations are used in this manuscript:

ASM	Artisanal and small-scale mining
HIV	human immunodeficiency virus
KAP	Knowledge, attitude and practices
OHS	Occupational Health and Safety
OSHA	Occupational Safety and Health Administration
PEL	Permissible exposure limits
PPE	Personal protective equipment
RMSE	Root mean square error
SADC	Southern African Development Community
SD	Standard deviations
Std err	Standard error
TB	Tuberculosis

## Appendix A

**Table A1:** Study knowledge questions asked study participants.

Question		Variable
Q1	Are you aware that there is a need for an occupational health and safety (H & S) policy in the workplace?	H&S policy knowledge
Q2	Do you know and understand the company	Health and safety policies?
Q3	Are you trained in Occupational Health and Safety hazards?	Training on OHS
Q4	Are you trained in occupational health and safety policy and related requirements? OHS policies	OHS policies
Q5	Does exposure to respirable crystalline silica cause lung cancer?	Lung cancer
Q6	Do you know the risks associated with high temperatures?	High temperature
Q7	Do you know the risks associated with carbon monoxide poisoning?	Carbon monoxide poisoning
Q8	Do you know the risks associated with nervous system problems?	Nervous system problems
Q9	Do you know the risks associated with hand-arm vibration?	Hand-arm vibration
Q10	Do you know the risks associated with the causes of silicosis?	Causes of silicosis
Q11	Do you know the risk associated with high noise exposure?	Levels of noise
Q12	Do you know the risks associated with chemical stressors?	Chemical stressors
Q13	Do you know the risks associated with biological agents?	Biological stressors
Q14	Do you know the risks associated with physical stressors?	Physical stressors
Q15	Do you know the risk associated with safety stressors?	Safety stressors
